# Chinese Soil Moisture Observation Network and Time Series Data Set for High Resolution Satellite Applications

**DOI:** 10.1038/s41597-023-02234-8

**Published:** 2023-07-01

**Authors:** Chunmei Wang, Xingfa Gu, Xiang Zhou, Jian Yang, Tao Yu, Zui Tao, Hailiang Gao, Qiyue Liu, Yulin Zhan, Xiangqin Wei, Juan Li, Lili Zhang, Lei Li, Bingze Li, Zhuangzhuang Feng, Xigang Wang, Ruoxi Fu, Xingming Zheng, Chunnuan Wang, Yuan Sun, Bin Li, Wen Dong

**Affiliations:** 1 Aerospace Information Research Institute, Chinese Academy of Sciences, 100094 Beijing, China; 2 National Engineering Research Center of Satellite Remote Sensing Applications, 100094 Beijing, China; 3 North China Institute is Aerospace Engineering, 065000 Langfang, China; 4 Northeast Institute of Geography and Agroecology, Chinese Academy of Sciences, 130102 Changchun, China; 5 College of Resources and Environment, University of Chinses Academy of Sciences, 100049 Beijing, China; 6 School of Geomatics and Prospecting Engineering, Jilin Jianzhu University, 130118 Changchun, China; 7 College of Geo-exploration Science and Technology, Jilin University, 130026 Changchun, China; 8 Changchun Jingyuetan Remote Sensing Test Site, Chinese Academy of Sciences, 130102 Changchun, China

**Keywords:** Hydrology, Hydrology

## Abstract

High-quality ground observation networks are an important basis for scientific research. Here, an automatic soil observation network for high-resolution satellite applications in China (SONTE-China) was established to measure both pixel- and multilayer-based soil moisture and temperature. SONTE-China is distributed across 17 field observation stations with a variety of ecosystems, covering both dry and wet zones. In this paper, the average root mean squared error (RMSE) of station-based soil moisture for well-characterized SONTE-China sites is 0.027 m^3^/m^3^ (0.014~0.057 m^3^/m^3^) following calibration for specific soil properties. The temporal and spatial characteristics of the observed soil moisture and temperature in SONTE-China conform to the geographical location, seasonality and rainfall of each station. The time series Sentinel-1 C-band radar signal and soil moisture show strong correlations, and the RMSE of the estimated soil moisture from radar data was lower than 0.05 m^3^/m^3^ for the Guyuan and Minqin stations. SONTE-China is a soil moisture retrieval algorithm that can validate soil moisture products and provide basic data for weather forecasting, flood forecasting, agricultural drought monitoring and water resource management.

## Background & Summary

Soil moisture is an important parameter in hydrological, climate prediction, drought monitoring and crop yield estimation models, as well as an important data source for research on global climate change and land surface data assimilation^1,2^. Therefore, accurate monitoring of soil moisture is of great significance. Traditional methods used to obtain soil moisture, such as the drying method and resistance method, can accurately measure the soil moisture value at a single point; however, the measurement accuracy is easily affected by human activities, weather conditions and monitoring range, and the measurement results at a single point do not meet application requirements. Therefore, accurate, timely and long-term ground observations of large-scale soil moisture can reveal trends in the water cycle in relation to climate or land cover change^3–6^.

With the development and progress of satellite remote sensing technology, soil moisture monitoring technology based on satellite visible-near-infrared and thermal infrared remote sensing data, active microwave and passive microwave have also been developed, which has greatly improved soil moisture monitoring capabilities. However, the soil moisture retrieved by satellite remote sensing on the surface is not as accurate as expected, and the low accuracy of products and inconsistency in standards and specifications among different products fails to meet user requirements, greatly limiting the practical value of remote sensing products^3^. Therefore, station-based measurements of soil moisture, which can represent the true value at the pixel scale and characterize the temporal dynamics, are the key to calibrating and validating satellite-based soil moisture retrievals^3,4,6^.

Due to the high cost of constructing long-term soil moisture measurement networks and an insufficient understanding of the importance of soil moisture in climate simulations and regional weather predictions, long-term soil moisture measurement networks are very scarce^4,7–9^. In the early 1990s, several specific soil moisture field surveys were conducted by the former Soviet Union, the United States, Europe, and Australia^9^,^10^, and until the 2000s, soil moisture monitoring networks were established as part of hydrological and meteorological observation facilities. With the launch of the Soil Moisture Ocean Salinity (SMOS) mission and the Soil Moisture Active Passive (SMAP) mission, many new soil moisture networks have emerged and been used to develop soil moisture algorithms and for product validation^10^,^11^.

The International Soil Moisture Network (ISMN) was launched in 2010, and it accesses soil moisture observation network data from many countries. With rich data sources and a large data volume, it is the largest soil moisture database in the world at present. The ISMN stores surface soil moisture data as well as deep soil moisture content and related hydrometeorological parameters, such as precipitation and soil temperature. Currently, satellite soil moisture product calibration, validation and algorithm improvement are the main application directions of the ISMN^3^. By November 2022, 72 ISMN soil moisture observation networks were connected, with a total of 2,451 sites (https://ismn.geo.tuwien.ac.at/en/).

In the past 20 years, many soil moisture observation networks have been established for remote sensing applications in China. In 2010, the Qinghai-Tibetan Plateau (QTP) observatory was the first soil water observation network established in China to study the calibration/validation of satellite- and model-based soil moisture products at a regional scale. This observatory includes the Naqu, Maqu, and Ngari networks^6^,^12^-^15^, and these station-based data have been used to quantify uncertainties in coarse-resolution satellites and models^16^. An ecohydrological wireless sensor network was established in the Heihe River Basin (HRB) in the arid region of Northwest China in 2012. The network is designed to capture the multiscale spatial variations and temporal dynamics of soil moisture, soil temperature, and land surface temperature simultaneously in heterogeneous farmland to provide remote sensing ground-truth estimates at an approximately kilometre-pixel scale using spatial upscaling^17^. The Dehui farmland soil observation network started in 2016 and was established by the Changchun Jingyuetan remote sensing test site, Chinese Academy of Science (CAS). Twenty-eight sensors are installed in the spatial range of 36 km × 36 km and are used to measure 0–5 cm soil moisture and soil temperature^18^,^19^. The Shandian River soil observation network was established in July 2018, and it covers an area of approximately 10,000 km^2^. The goal of this network is to facilitate studies of water and energy exchanges between the land and atmosphere at a regional scale as well as provide a long-term ground reference for validating various satellite products. There are three sampling scales: 100 km (large scale), 50 km (medium scale), and 10 km (small scale)^20^. The Genhe soil water observation network was established in 2013. The network monitored soil moisture and temperature at depths of 3, 5, 10 and 20 cm^21^, which were used to validate the AMSR-2, SMOS, and SMAP soil moisture products. The Henan Soil Water Monitoring Network was established in 2009 in Henan Province, China. It covers nearly all of Henan Province, with a total area of 16.7 × 104 km^2^ ^22^.

With the development of soil moisture networks, much basic theoretical and applied research progress has been made; however, the existing observation data are still inadequate, such as inconsistency among sensors and soil depths, various sampling distributions are used, and measurements are only taken at the regional scale. These limitations will seriously affect the application of datasets at the national scale^23^-^26^. On the other hand, with the launch of high spatial resolution satellites at home and abroad in recent years, especially GaoFen series and space-based system satellites launched by China, there is an urgent need to build a national and uniform standards network aimed at the validation and development of high-resolution satellite remote sensing products. An automatic soil observation network in typical ecosystems in China (SONTE-China) was established by the Aerospace Information Research Institute (AIR), Chinese Academy of Sciences (CAS), which had a unified sensor, unified sample design, unified acquisition depth, unified calibration method, and unified data processing method. SONTE-China can automatically obtain *in situ* soil moisture data in real time and at spatial scales up to the pixel scale. SONTE-China covers the main ecosystems (including grassland, farmland, desert and forest) and dry/wet climate zones in China, and it can develop and validate high-resolution satellite remote sensing soil moisture products and provide basic data for research in the fields of weather forecasting, flood forecasting, agricultural drought monitoring and water resource management.

This paper reports on the status of SONTE-China and presents pixel-scale soil temperature and soil moisture at 17 typical stations from approximately 2019 to 2021. The 3-year soil moisture dataset of SONTE-China includes the continuous daily mean data at the pixel scale obtained at four soil depths (5 cm, 10 cm, 20 cm and 40 cm) using the arithmetic average spatial upscaling method^6^,^12^.

This paper is organized as follows. Section 2 describes the status of SONTE-China and the *in situ* SM measurements, as well as the method of sensor calibration and image data processing. Section 3 introduces the data records. Section 4 presents the soil moisture calibration analysis, temporal and spatial changes in soil moisture and soil temperature, and the relationship between soil moisture and Sentinel-1 radar data.

## Methods

### Status of SONTE-China

**Fig. 1:**
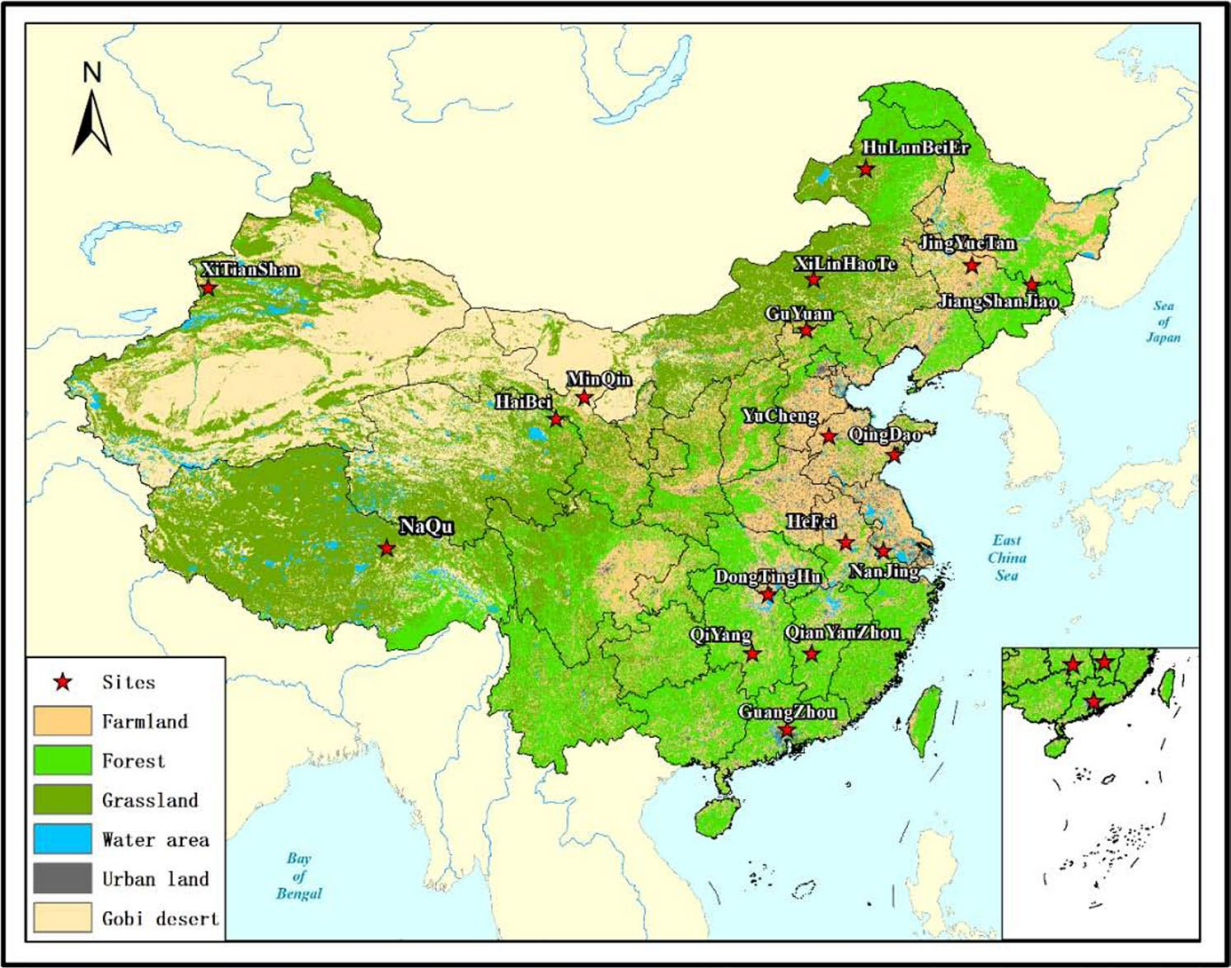
The geographical locations of 17 observation stations and their main coverage types in China. The names (abbreviations) are JingYueTan (JYT), GuYuan (GY), HeFei (HF), HaiBei (HB), JiangShanJiao (JSJ), XiTianShan (XTS), MinQin (MQ), HuLunBeiEr (HLBE), XiLinHaoTe (XLHT), QiYang (QY), DongTingHu (DTH), GuangZhou (GZ), YuCheng (YC), NanJing (NJ), QingDao (QD), QianYanZhou (QYZ), and NaQu (NQ).

**Fig. 2:**
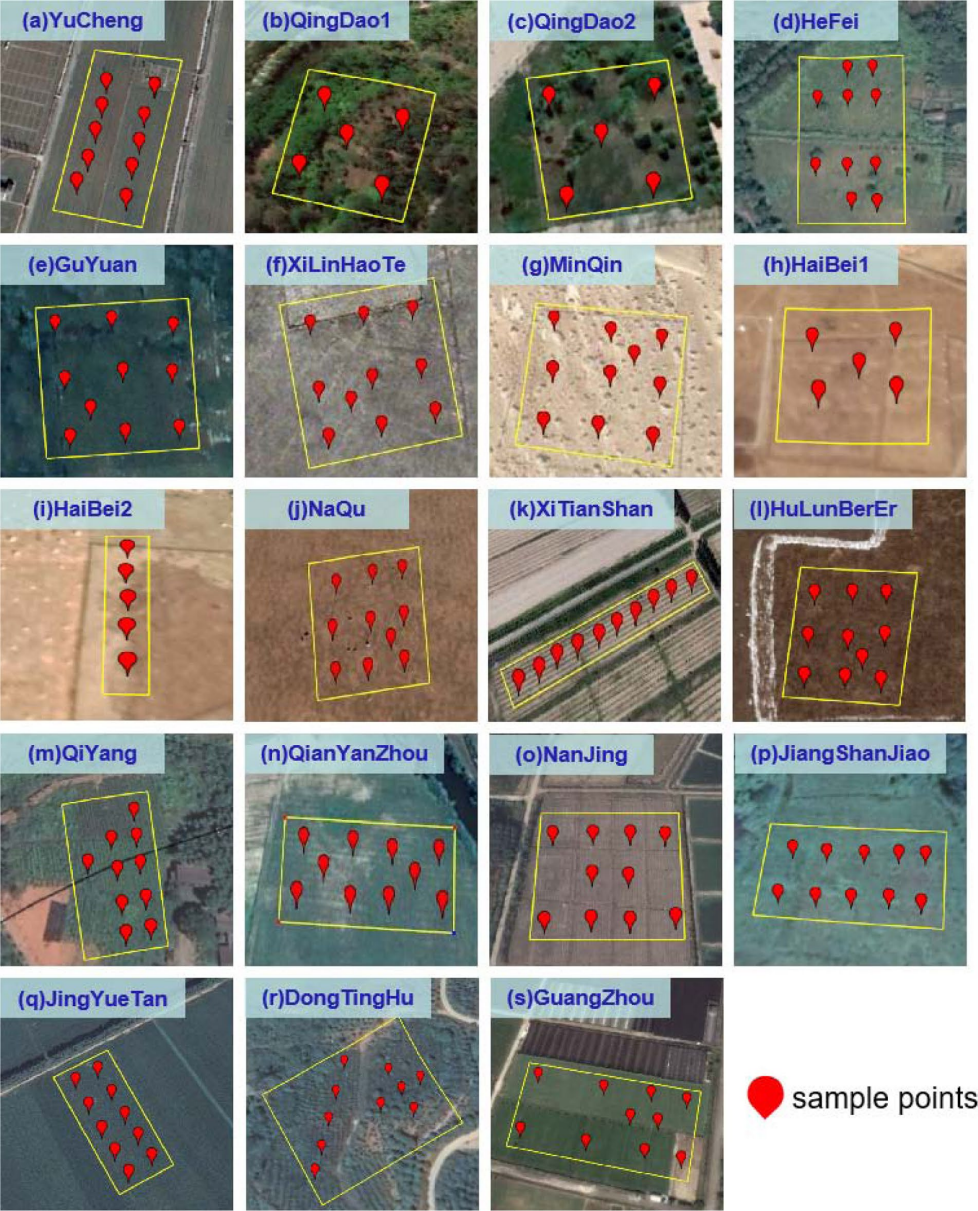
Spatial distribution of the 5TM soil sensor for all 17 sites in SONTE-China. There are two instrument installation areas (QingDao1 and QingDao2, HaiBei1 and HaiBei2) for the site, and four layers of sensors are deployed at each site.

A soil observation network in typical ecosystems in China (SONTE-China) was established to monitor the changes in soil temperature and moisture in different ecosystems and provide an accurate pixel-scale ground observation dataset. Seventeen SONTE-China stations are located in 13 provinces across China (Fig. 1); the longitude range is 81.18°~128.96°E, the latitude range is 23.24°~49.34°W, and it covers a variety of ecological types, including grassland, farmland, desert and forest. Each station covers a uniform area of approximately 1 ha, and soil moisture and soil temperature were measured by a Metre 5TM sensor with a 30-minute measurement interval. Each station consists of 10 observation nodes. Each observation node contains four observation soil depths (5 cm, 10 cm, 20 cm and 40 cm). The spatial distribution of the 5TM soil sensor for all 17 SONTE-China stations is given in Fig. 2. Supplementary Table 1 provides information on the geographic locations, crop types, soil properties and climatic conditions for the 17 sites.

Soil samples were collected at four different depths (0–5 cm, 7.5–12.5 cm, 17.5–22.5 cm and 37.5–42.5 cm) at 17 stations for calibration experiments and laboratory analysis. The results showed that the percentages of clay, silt and sand ranged from 1.09% to 20.94%, 10.71% to 85.42% and 3.29% to 87.94%, respectively (Table 1). The content ranges of soil organic matter (SOM) and salinity are 0.17%–17.45% and 0.003%–1.73%, respectively. Obviously, the SOM tends to be high at the surface and decreases with increasing depth, whereas the soil salinity content is the opposite (Table 1). Jiangshanjiao station has the highest SOM of the 0–40 cm layer but the lowest salinity content among all stations.

**Table 1:** Range of different soil properties at four different depths at 17 SONTE-China stations.

Soil depth range (cm)	Clay range (%)	Silt range (%)	Sand range (%)	SOM range (%)	Salinity range (%)
0–5	1.34–16.79	10.72–84.20	5.20–87.94	0.18–17.46	0.01–0.28
7.5–12.5	1.09–19.31	13.31–85.43	3.30–85.60	0.22–15.62	0.01–0.16
17.5–22.5	1.18–19.89	14.27–84.49	3.68–84.55	0.26–11.65	0.01–0.22
37.5–42.5	2.90–20.94	23.53–84.46	4.80–73.57	0.26–5.38	0.01–0.31

### Observation dataset acquisition

**Fig. 3:**
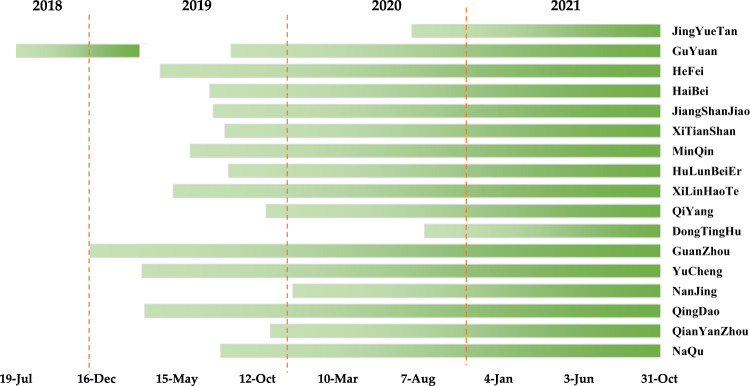
Observation start time for 17 stations in the soil observation network in typical ecosystems in China (SONTE-China). There are data missing at the GuYuan site due to instrument maintenance.

The SONTE-China dataset includes soil moisture and soil temperature observation data from 17 stations. Figure 3 shows the measurement start times of all 17 stations. GuYuan station began obtaining observations in July 2018, the earliest station to do so, and the latest station to begin observations was DongTingHu station in August 2020. Currently, the data acquisition time of all stations has exceeded 2 years, and the data will be provided continuously in the future.

The purpose of the SONTE-China dataset is to provide daily mean values for the site at the pixel scale for high-resolution satellite applications. Therefore, spatial upscaling is usually necessary to obtain the regional-scale value of an *in situ* network from multiple monitoring sites to match the footprint of high-resolution satellite products. The frequently used approach is the arithmetic average^6^,^12^. The arithmetic averaging method assigns an equal weight coefficient to each observation node of the station, which can be formulated as

1 $${\mathop{\theta }\limits^{\leftharpoonup }}_{t}^{ups}=\frac{1}{N}{\sum }_{i=1}^{N}\frac{1}{T}{\sum }_{t=1}^{T}{\theta }_{t,i}^{obs}$$

where *i* represents the *i*th observation node, N represents the total number of observation nodes (*N* = 10), and t is the number of observations per day (*T*=48, observation frequency of 30 minutes). ${\bar{\theta }}_{t}^{ups}$ represents the upscaled soil moisture/soil temperature, and ${\theta }_{t}^{obs}$ represents soil moisture/soil temperature measurements.

### Soil moisture sensor calibration

Soil moisture sensors are the main method for measuring soil moisture, and their measurement datasets can be used as standard datasets for evaluating the results from remote sensing and data assimilation. The spatial changes in soil properties (texture, SOM, and salinity) affect the dielectric constant measured by soil moisture sensors and the soil moisture accuracy. Therefore, it is necessary to consider the effects of soil properties to obtain high-accuracy soil moisture measurements for SONTE-China. The capacitance-based soil moisture sensor (5TM, Decagon Devices, Inc.) is used to measure the dielectric constant of soil. The specific working principles of the 5TM sensor can be found in the literature^27^. The soil moisture calibration experiment was designed to improve the measurement accuracy of the 5TM sensors by simulating different soil moisture contents (from dry to saturation). The volume of soil samples used in the calibration processes was approximately 0.03 m^3^ per station after pretreatment, and the volume used to obtain the true values by the gravimetric method was approximately 0.006 m^3^. The calibration processes are described as follows: (1) Soil samples were passed through a 5 mm sieve to remove structures, such as rocks and plants. Soil samples were oven-dried at 75 °C until constant weight. (2) Approximately 3% water by weight of the soil sample from step (1) was added and homogenously mixed. (3) Using three 5TM sensors, 5–6 measurements were recorded, and the average measurement was taken as the result. (4) Soil samples were collected by ring knife from the position where the sensors were inserted and, the “true” soil moisture value was measured using the gravimetric method. (5) Soil was added to maintain a constant weight of the measured soil samples. The weight of the added soil samples was equal to the weight of the gravimetric samples. (6) The above measurements were repeated until the soil sample was saturated. Station-based soil samples from 4 depths at 17 sites were used in the calibration experiments, and each soil sample was measured 7–12 times due to differences in soil texture.

**Fig. 4:**
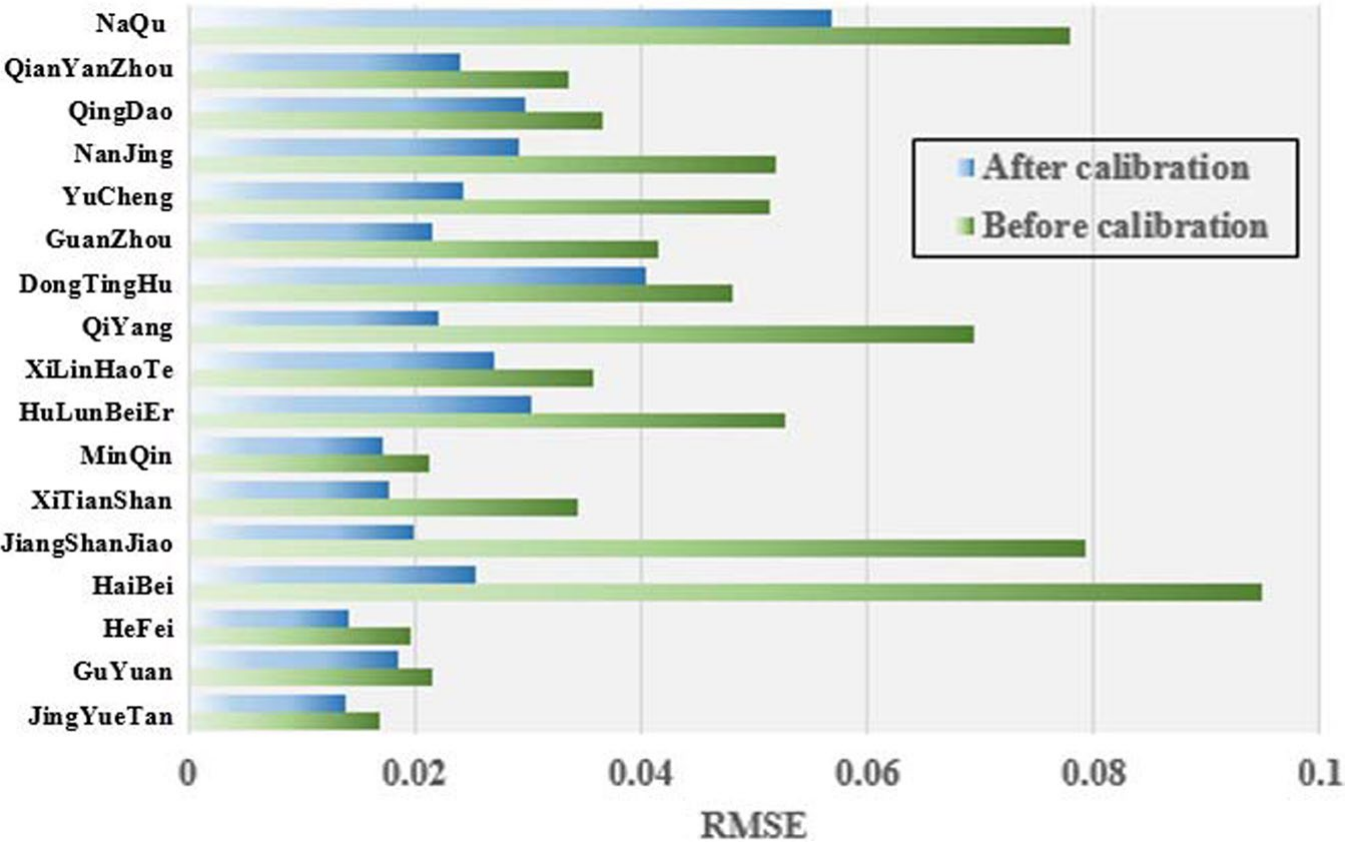
Comparison of root mean square error (RMSE) (unit: m3/m3) values of 5TM soil moisture for 17 SONTE-China stations before and after calibration for station-based soil types.

The soil moisture values measured by the 5TM sensor (*θ*_5TM_) and the gravimetric method measurements (*θ*_true_) were obtained after the calibration experiments. *θ*_5TM_ was underestimated, but there were good linear relationships between *θ*_5TM_ and *θ*_5true_ for all 17 stations with high correlation coefficients (Fig. 4). Therefore, a linear calibration model (LCM) was reasonable for calibrating *θ*_5TM_. The calibration process for the LCM is described as follows: (1) the coefficients of the LCM (a_LCM_ and b_LCM_) were determined by integrating *θ*_5TM_ and *θ*_true_ into Eq. 2. Each soil sample had its own calibration coefficient; (2) the soil moisture of the LCM (*θ*_LCM_) was calculated using Eq. 3, and the coefficients a_LCM_ and a_LCM_ were obtained from Eq. 2:

2 $${\theta }_{true}(i,j,k)={a}_{LCM}(i,j)\times {\theta }_{5TM}(i,j,k)+{b}_{LCM}(i,j)$$

3 $${\theta }_{LCM}(i,j,k)={a}_{LCM}(i,j)\times {\theta }_{5TM}(i,j,k)+{b}_{LCM}(i,j)$$

where *a*_*LCM*_ and *b*_*LCM*_ are coefficients in the LCM; *I, j, k* represent different stations with a range of 1 to 17; soil samples at different depths with a range of 1 to 4; and soil samples with different *θ* in the calibration experiments with a range of 1 to 12. Please refer to Li *et al*.^27^ for the specific calibration process and method.

### High-resolution satellite soil moisture product inversion

The radar and optical images with 10 m spatial resolution used here are from the sentinel-1 and sentinel-2 satellites of the ESA (https://scihub.copernicus.eu/dhus/#/home). The data are downloaded based on the GEE platform. Sentinel-1 (IW mode) C-band radar data were preprocessed by orbit correction, radiometric calibration, multilook, filtering and terrain correction. Sentinel-2 data are pretreated with radiometric correction and atmospheric correction, and finally, the NDVI (Normalized Difference Vegetation Index) dataset is generated based on GEE. All preprocessed remote sensing data (Sentinel-1&2) were extracted according to the boundaries of the station, and the average of the remote sensing data (with multiple pixel values) in the extracted station range was taken as the effective station satellite observational data for soil moisture inversion.

The sensitivity of the radar signal to soil moisture decreases in the frozen soil period. It is assumed that 0 °C is the threshold of the frozen soil period, and long-term soil moisture data below 0 °C are eliminated. Sentinel-1 (GuYuan site: 91 scenes and MinQin site: 138 scenes) IW mode C-band radar data are adopted to analyse its relationship with station-based soil moisture. A cosine normalization model is used to normalize the incident angle effect of radar data^28^,^29^. The incidence angle ranges of the radar data for the GuYuan site and MinQin site are 31° to 42° and 35° to 45°, respectively, and 35° and 40° are selected as the normalized reference angles. The NDVI of the Sentinel-2 data caused by cloud contamination has abnormal and missing values, and S-G filtering is used for reconstruction of the NDVI^30^.

The vegetation canopy has a direct influence on radar backscattering, which affects the accuracy of the SAR soil moisture retrieval. The water cloud (W-C) model was used to eliminate the effect of the vegetation canopy on radar signals^31^-^34^.

4 $${\sigma }_{pp}^{0}={\sigma }_{veg}^{0}+{\delta }^{2}{\sigma }_{soil}^{0}$$

5 $${\sigma }_{veg}^{0}=A\cdot {V}_{1}\cdot \cos \theta \cdot \left(1-{\delta }^{2}\right)$$

6 $${\delta }^{2}=\exp \left(-2B{V}_{2}\sec \theta \right)$$

where ${\sigma }_{soil}^{0}$ represents the scattering term of soil; ${\sigma }_{veg}^{0}$ represents the scattering term of vegetation; *δ*^2^ is the two-layer attenuation coefficient of vegetation; and *V*_*1*_ and *V*_*2*_ represent the description factors of the vegetation canopy, which are expressed by the NDVI in this paper. A and B are empirical coefficients.

After S-G filtering of the NDVI time series, incidence angle correction and vegetation correction based on the W-C model, ${\sigma }_{soil}^{0}$ is obtained under a uniform incident angle. For regions with small surface roughness, simple linear relationships can be used to retrieve soil moisture. For areas with medium-high roughness, an exponential function can better describe the relationship between soil moisture and σ° ^35^. The simple regression model based on the exponential relationship is given as follows:

7 $$SM=\exp \left(C\ast {\sigma }_{soil}^{0}-D\right)$$

where C and D are empirical coefficients. D can represent the ${\sigma }_{soil}^{0}$ variation resulting from surface roughness.

## Data Records

We have completed major procedures, including network design, sensor installation, data collection, sensor calibration, pixel scale data, and data archive. All data were quality controlled by checking soil moisture time series one by one to remove suspected or wrong data. The metadata include station geographic position, elevation, land cover, and soil texture, as well as SOC content and start time.

This dataset provides the daily mean data at the pixel scale of 17 typical ecosystem sites through the end of 2021. The SONTE-China dataset presented in this article is available at the following link: 10.6084/m9.figshare.21302955.v2. The dataset is stored in EXCEL format^36^.

## Technical Validation

### Soil moisture calibration for station-based soil types

According to the soil samples corresponding to the buried soil depth of the 5TM sensor collected in the field, laboratory calibration of the 5TM sensor was carried out. The RMSE of the 5TM soil moisture measurement before and after calibration for specific soil types is shown in Fig. 4. Before calibration, the maximum, minimum and average RMSEs are 0.095 m^3^/m^3^, 0.019 m^3^/m^3^ and 0.049 m^3^/m^3^, respectively, and their values become 0.057 m^3^/m^3^, 0.014 m^3^/m^3^ and 0.027 m^3^/m^3^ after calibration, respectively. Obviously, the accuracy of the 5TM soil moisture is significantly improved after calibration for station-based soil types.

**Fig. 5:**
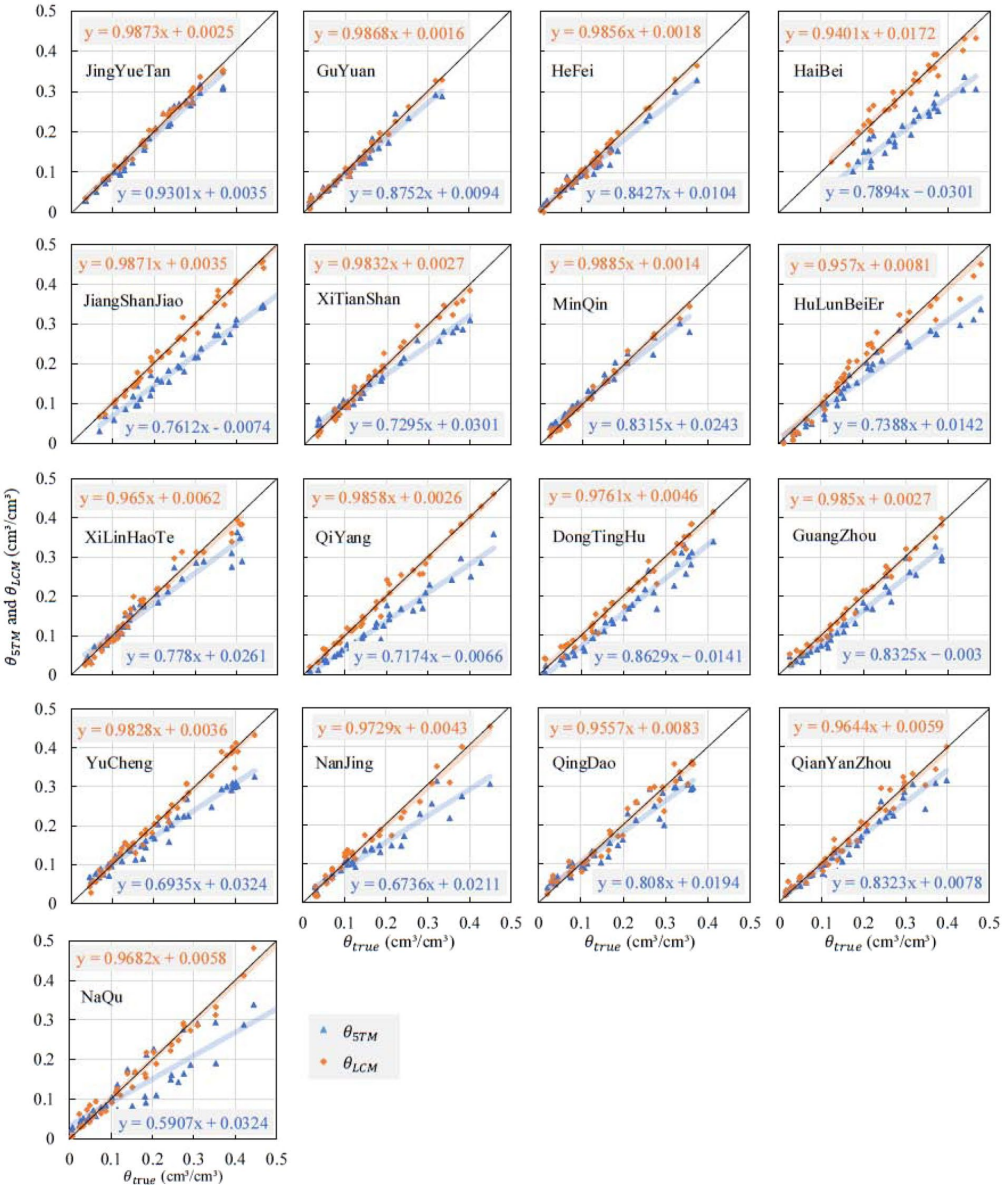
Comparison of *θ*_true_ with the sensor-based soil moisture (*θ*_5TM_ and *θ*_LCM_) for all stations and all four soil depths^25^.

The calibration results indicated that *θ*_5TM_ was significantly underestimated, which was demonstrated by a total of 611 soil sample datasets from 17 sites (Fig. 5). The underestimation of *θ*_5TM_ increases with soil moisture, and only 39.7% of the *θ*_5TM_ results achieve a factory accuracy of ±0.03 m^3^/m^3^. After calibrating *θ*_5TM_ based on the LCM, experimental datasets from 17 sites showed an improvement in average *R* from 0.95 to 0.99 and a decrease in average *RMSE* from 0.049 to 0.027 m^3^/m^3^.

**Fig. 6:**
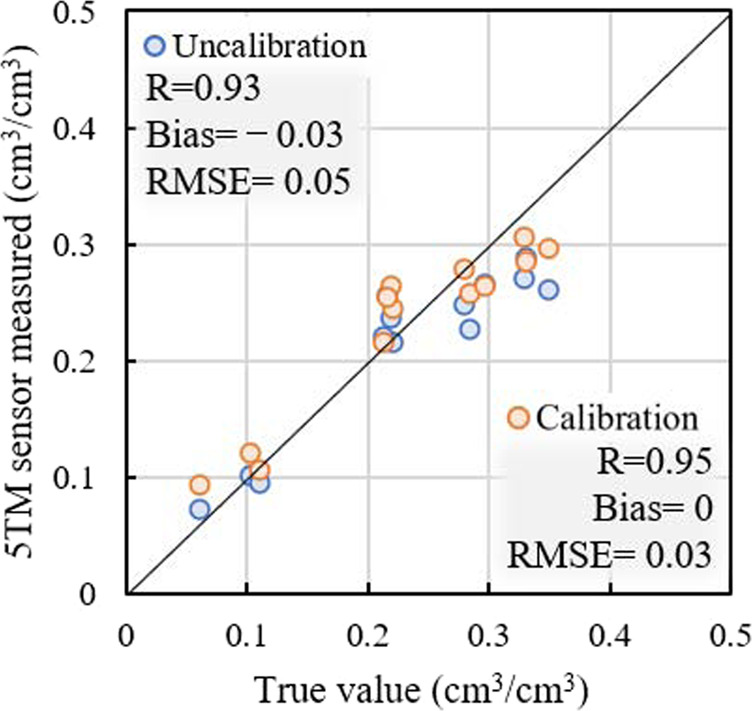
Comparison of calibrated/uncalibrated soil moisture values with true values at the JinYueTan, XiLinHaoTe and YuCheng sites.

To further verify the soil moisture accuracy of SONTE-China, the calibrated soil moisture (*θ*_LCM_) was compared with field soil moisture. Figure 6 shows the comparison of the calibrated/uncalibrated soil moisture with the measured values at the JinYueTan, XiLinHaoTe and YuCheng sites. After calibration, the correlation (R) improved from 0.93 to 0.95, and the RMSE decreased from 0.05 to 0.03 m^3^/m^3^. Obviously, the calibrated soil moisture weakens the underestimation problem of SONTE-China soil moisture, especially for high soil moisture.

### Temporal and spatial changes in station-based soil moisture and soil temperature

**Fig. 7:**
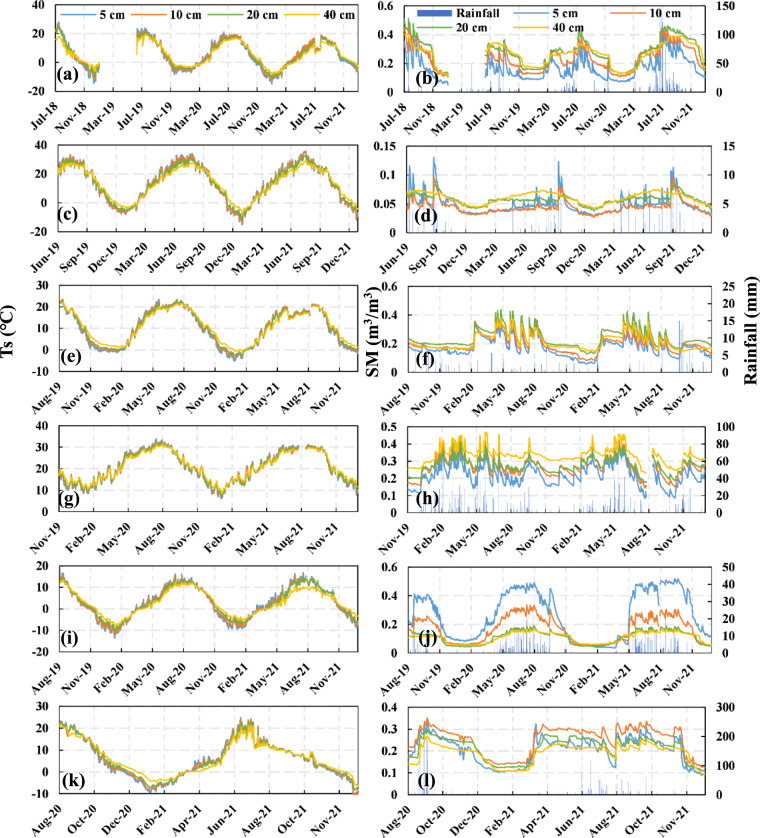
Temporal changes in soil moisture and soil temperature at multiple observed soil depths. (**a,b**) Guyuan, (**c,d**) Minqin, (**e,f**) Xitianshan, (**g,h**) Qianyanzhou, (**I,j**) Naqu, (**k,l**) Jingyuetan.

The SONTE-China data are distributed across different climatic zones and land cover types in China. According to the geographical location and land cover type, six stations (Guyuan, Minqin, Xitianshan, Qianyanzhou, Naqu and Jingyuetan) were selected to analyse the temporal and spatial changes in soil moisture and soil temperature (Fig. 7). There was strong seasonality for the multilayer soil temperature observed at six representative stations. The soil temperature is high in summer and low in winter, and the seasonality of a single site over the years is basically stable. This result is consistent for multiple soil depths. Taking the soil temperature at depths of 5 cm and 40 cm at the Jingyuetan site as an example, in summer, the soil temperature at a depth of 5 cm was higher than that at a depth of 40 cm; in winter, the opposite was true.

The soil moisture observed at each station showed seasonality (Fig. 7). The soil moisture observed in summer is generally higher than that observed in winter. Compared with deep soil moisture, shallow soil moisture has a larger amplitude due to the difference in precipitation and evapotranspiration. For example, the soil moisture at a depth of 5 cm at the Minqin station has a small number of observations with high variation, which may be related to rainfall/artificial irrigation. In addition, the soil moisture value of the station shows an increasing trend with soil depth. Notably, Naqu shows the opposite behaviour, and the moisture value of the 5 cm soil surface is the wettest. The main reason is that there is a layer of organic matter in the soil surface of the Naqu site, which can maintain water^11^.

Here, the spatial change in soil temperature and soil moisture was investigated from the observed values of the 17 SONTE-China stations at the 0–5 cm soil depth (Fig. 7). In terms of soil temperature, the observed data in northern China are generally lower than those in southern China. For example, the median temperature at the Hulunbeier site (1.66 °C), a typical site in the north, is the lowest value of the 17 sites, and the median temperatures of Guyuan and Jingyuetan are lower than 5 °C. The median temperature of typical sites in the south (such as Guangzhou and Qianyanzhou) is higher than 20 °C, and the median temperature of the Guangzhou site (22.41 °C) is the highest. This finding is consistent with the typical characteristics of the soil temperature distribution in the middle and high latitudes of the Northern Hemisphere, and the soil temperature decreases with increasing latitude. In addition, Haibei and Naqu have lower median temperatures, which are 3.22 °C and 3.48 °C, respectively. The two sites are located on the QTP at high altitudes. Topographic elevation becomes the main factor affecting soil temperature.

**Fig. 8:**
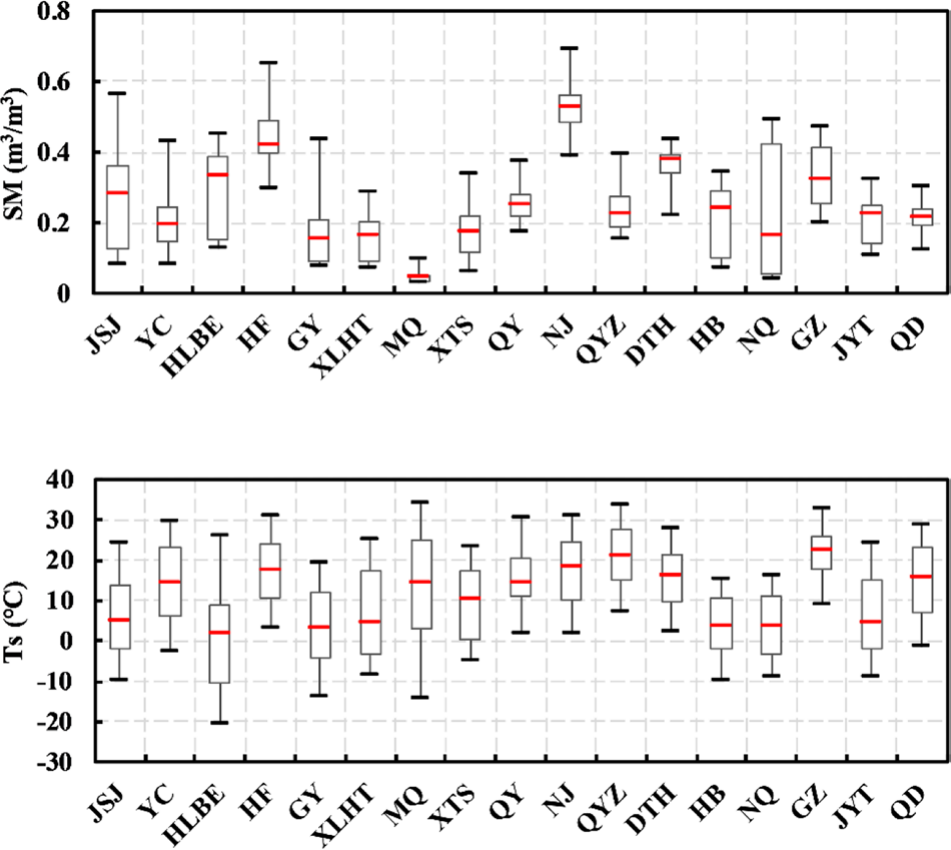
Statistical values (maximum, minimum and median) of soil moisture and soil temperature at the 0–5 cm soil depth among all 17 observation sites from August 30, 2020, to July 30, 2021.

In terms of soil moisture, the southern site is generally wetter, and the northern site is relatively drier (Fig. 8). For example, the highest value of median soil moisture at 17 sites appears at the Nanjing site (0.53 m^3^/m^3^), and the median soil moisture of Hefei and Dongtinghu at typical southern sites is also high, at 0.42 m^3^/m^3^ and 0.37 m^3^/m^3^, respectively. The minimum value of median soil moisture appears in the typical northern site of Minqin, with a value of 0.05 m^3^/m^3^. The median soil moisture values of Guyuan (0.15 m^3^/m^3^) and Xilinhaote (0.16 m^3^/m^3^) are also low. This finding is consistent with the characteristics of dry and wet climate zones in China. Notably, Minqin belongs to a temperate continental arid climate area with less precipitation and a dry climate. The annual soil moisture difference in Minqin is only 0.07 m^3^/m^3^.

### Applications for radar soil moisture inversion

**Fig. 9:**
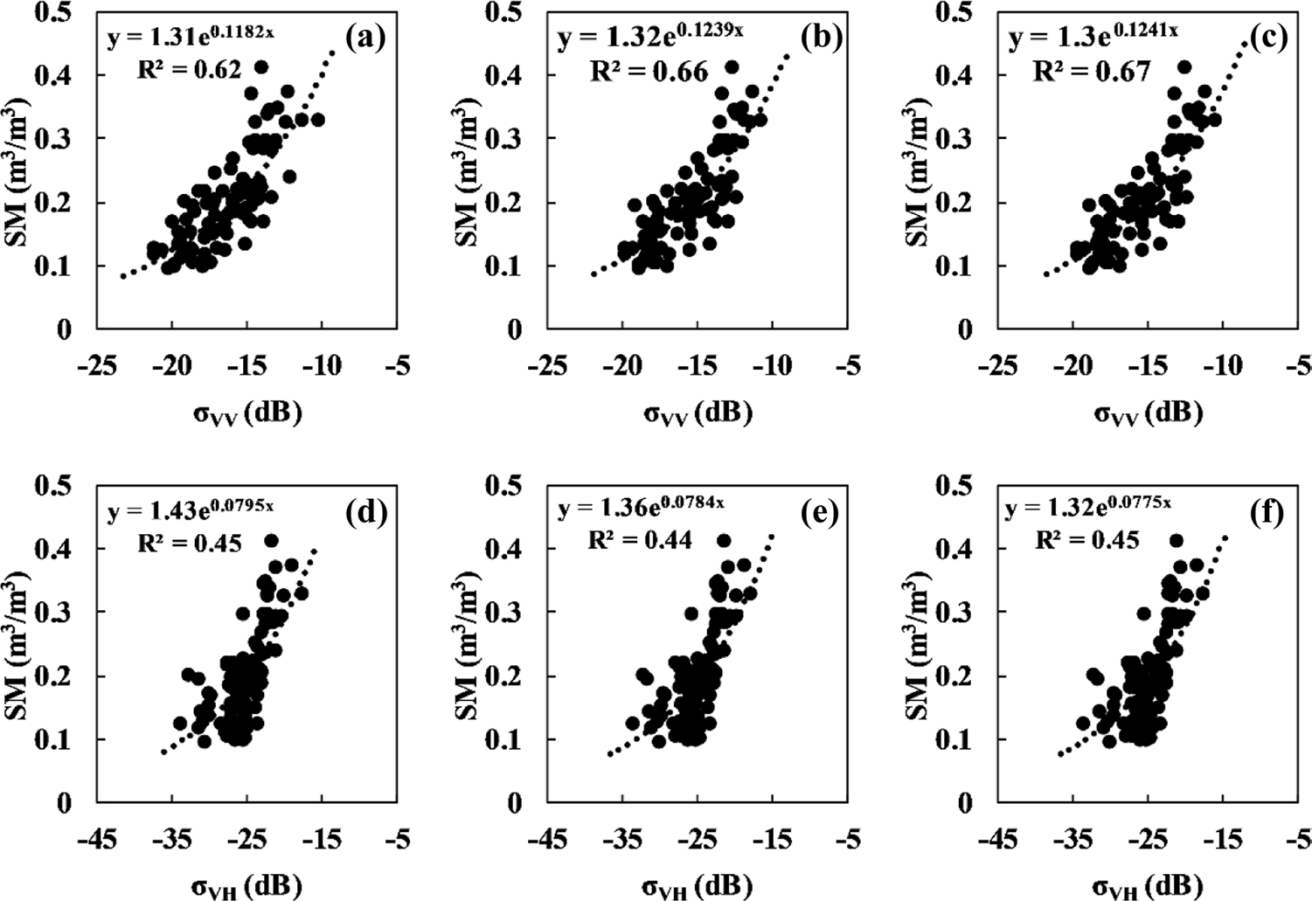
Relationship between the Sentinel-1 C-band radar backscattering coefficient and soil moisture for the GuYuan station. (**a**) and (d) denote the original σ_VV_ and σ_VH_, (**b**) and (**e**) denote σ_VV_ and σ_VH_ after incidence angle correction, and (**c**) and (**f**) denote σ_VV_ and σ_VH_ after incidence angle and vegetation correction.

**Fig. 10:**
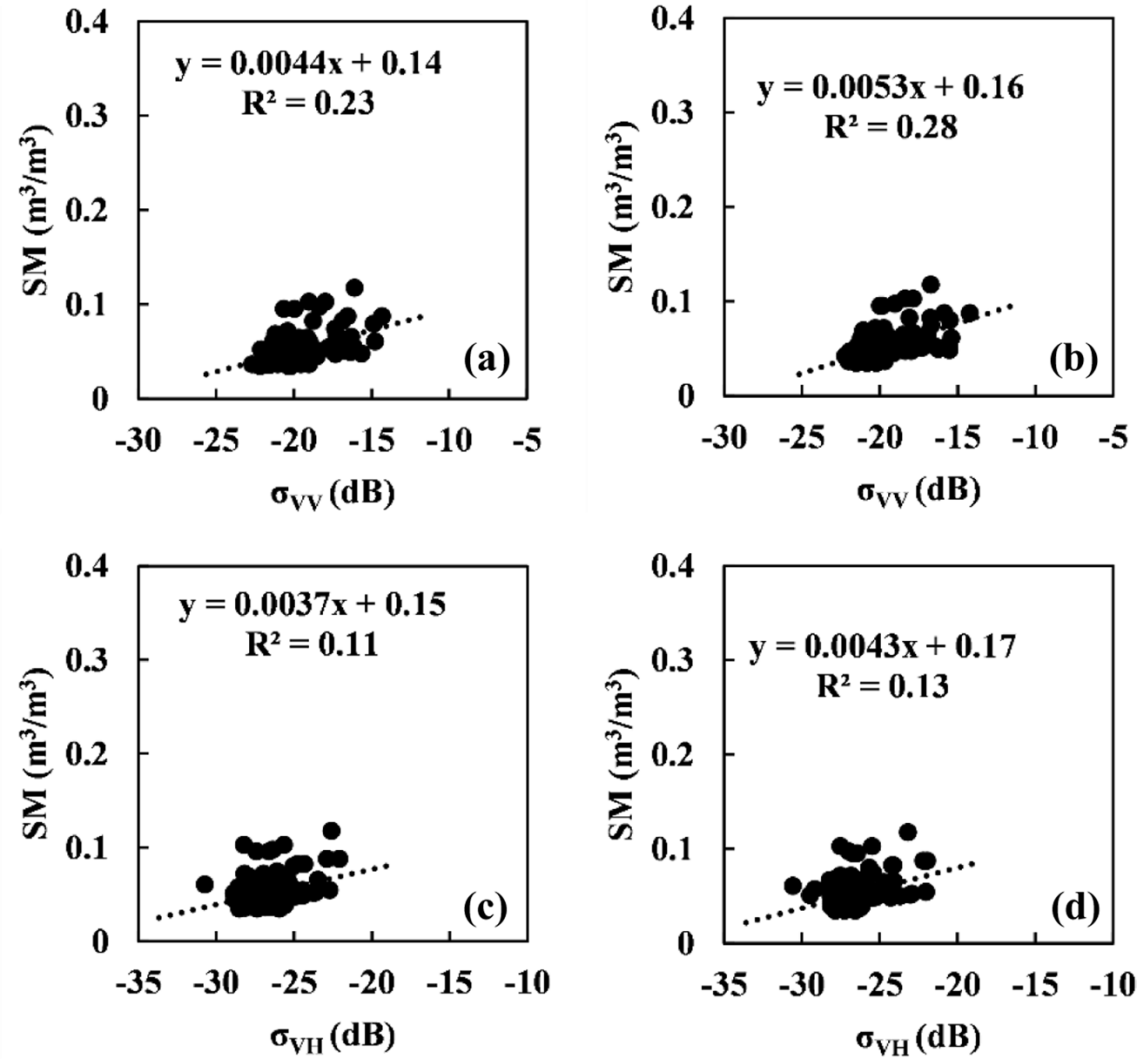
Relationship between the Sentinel-1 C-band radar backscattering coefficient and soil moisture for the MinQin station. (**a**) and (**c**) denote the original σ_VV_ and σ_VH_, and (**b**) and (**d**) denote σ_VV_ and σ_VH_ after incidence angle correction.

Radar remote sensing is usually used for soil moisture estimation because of its sensitivity to soil moisture and penetration of vegetation canopies. By analysing the correlation between the measured soil moisture and Sentinel-1 radar backscattering coefficient, on the one hand, the effectiveness of SONTE-China at obtaining soil moisture is observed; on the other hand, the feasibility of estimating surface soil moisture using radar data is observed. Here, the station-based soil moisture of the 0–5 cm soil depth at the GuYuan site (July 2018 to July 2021) and the MinQin site (June 2019 to August 2021) are considered to be representative application cases. Figures 9, 10 show the correlation between radar signals and station-based soil moisture at the GuYuan station and MinQin station.

At the GuYuan station, the original radar signal has a good correlation with the measured soil moisture (R^2^ = 0.62) under VV polarization. The correlation between the radar signal after angle correction and the measured soil moisture (R^2^ = 0.66) is further improved. The correlation between radar signals after angle correction and vegetation correction and measured soil moisture is better than that with only angle correction, and R^2^ is 0.67. The correlation between the original radar signal and the measured soil moisture under VH polarization (R^2^ = 0.45) is good overall but slightly worse than that under VV polarization. The correlation between the angle-corrected radar signals and soil moisture was not significantly improved, nor was the correlation between the angle-corrected and vegetation-corrected radar signals and soil moisture.

Compared with the GuYuan station, the correlation between soil moisture and the radar signal at the MinQin station is poor, with R^2^ values of 0.23 and 0.11 under VV and VH polarizations, respectively. The MinQin station has a continental desert climate, and its sand content reaches 87.94%. The water holding capacity of the soil is poor. The relatively low variation range of soil moisture at the MinQin station results in the same small response range of the corresponding radar signal. The effect of the vegetation canopy on the radar signal at MinQin Station can be ignored, so the W-C model is not used to remove the vegetation effect at this station. The correlation between the radar signal and soil moisture was further improved after correction of the incident angle effect, and the *R*^2^ values of VV and VH polarizations were 0.28 and 0.13, respectively.

**Fig. 11:**
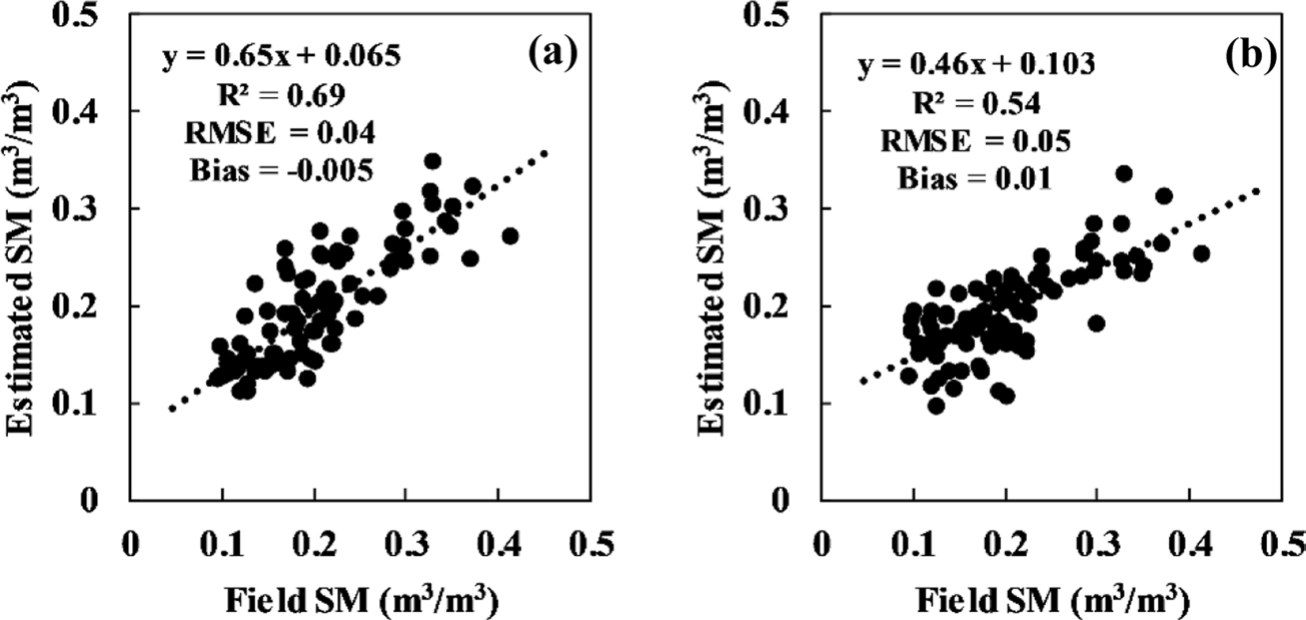
Comparison of station-based soil moisture and retrieved soil moisture from the Sentinal-1 C-band radar backscattering coefficient after correcting the incidence angle and vegetation for the GuYuan station. (**a**) VV polarization, (**b**) VH polarization.

**Fig. 12:**
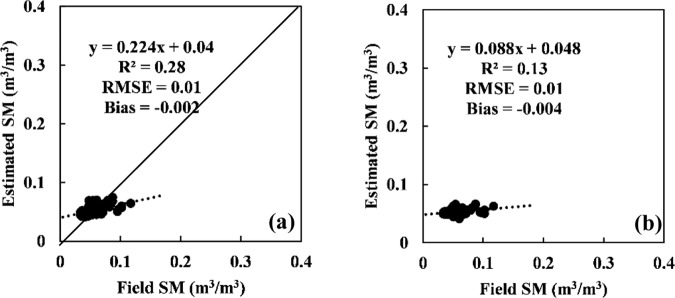
Comparison of station-based soil moisture and retrieved soil moisture from the Sentinel-1 C-band radar backscattering coefficient after correcting the incidence angle for the MinQin station. (**a**) VV polarization, (**b**) VH polarization.

Based on the fitting relationship between the corrected radar signal and station-based soil moisture (Figs. 9c,f,10b,d), soil moisture can be computed. The comparison of station-based and computed soil moisture is given in Figs. 11, 12. A strong correlation was found for both VV and VH polarizations at the Guyuan station; the R^2^ values under VV and VH polarizations were 0.69 and 0.54, respectively, and the RMSE values were 0.04 m^3^/m^3^ and 0.05 m^3^/m^3^, respectively. At the Minqin station, the inversion and measured values of soil moisture have lower RMSEs, which are 0.01 m^3^/m^3^ under VV and VH polarizations.

### Supplementary information

Supplementary Table 1

## Data Availability

MATLAB scripts that implement normalization of incidence angle effect, vegetation removal, soil moisture inversion, and mapping are available at (https://figshare.com/articles/online_resource/Example_of_sm_inversion_zip/21376089). Further questions can be directed towards Chunmei Wang (wangcm@ aircas.ac.cn)
