# Author Correction: Chinese Soil Moisture Observation Network and Time Series Data Set for High Resolution Satellite Applications

**DOI:** 10.1038/s41597-023-02775-y

**Published:** 2023-12-15

**Authors:** Chunmei Wang, Xingfa Gu, Xiang Zhou, Jian Yang, Tao Yu, Zui Tao, Hailiang Gao, Qiyue Liu, Yulin Zhan, Xiangqin Wei, Juan Li, Lili Zhang, Lei Li, Bingze Li, Zhuangzhuang Feng, Xigang Wang, Ruoxi Fu, Xingming Zheng, Chunnuan Wang, Yuan Sun, Bin Li, Wen Dong

**Affiliations:** 1 Aerospace Information Research Institute, Chinese Academy of Sciences, 100094 Beijing, China; 2 National Engineering Research Center of Satellite Remote Sensing Applications, 100094 Beijing, China; 3 North China Institute is Aerospace Engineering, 065000 Langfang, China; 4 Northeast Institute of Geography and Agroecology, Chinese Academy of Sciences, 130102 Changchun, China; 5 College of Resources and Environment, University of Chinses Academy of Sciences, 100049 Beijing, China; 6 School of Geomatics and Prospecting Engineering, Jilin Jianzhu University, 130118 Changchun, China; 7 College of Geo-exploration Science and Technology, Jilin University, 130026 Changchun, China; 8 Changchun Jingyuetan Remote Sensing Test Site, Chinese Academy of Sciences, 130102 Changchun, China

**Keywords:** Hydrology, Hydrology

Correction to: *Scientific Data* 10.1038/s41597-023-02234-8, published online 01 July 2023

Qiyue Liu, North China Institute is Aerospace Engineering, was incorrectly omitted as an author of this work in the original version. This has been corrected via additions to the author list and author contributions statement in the HTML and pdf versions of the paper.

