# Supplementary material for: Chinese Soil Moisture Observation Network and Time Series Data Set for High Resolution Satellite Applications
Source: Sci Data. 2023 Jul 1;10:424. doi: 10.1038/s41597-023-02234-8 (PMC10314894; doi:10.1038/s41597-023-02234-8)
Supplement: Supplementary file 1 — Supplementary Table 1 [file 41597_2023_2234_MOESM1_ESM.docx]

Supplementary Table 1

The seventeen SONTE-China observation stations are located in 13 provinces across China (Figure 1). It covers a variety of ecological types, including grassland, farmland, desert and forest. Table S1 introduces the geographic location, crop type and climate condition information for all 17 stations.

Table S1 Geographic location, crop type and climate condition information for all 17 stations.

| Site name | Geographic location | Information about climate, soil, land cover |
| --- | --- | --- |
| JingYueTan  (JYT) | JingYueTan network is in Houling village, Kaoshan Town, Nong'an County, Changchun City, Jilin Province. This network is in the experimental area of the Changchun Jingyuetan remote sensing test station, CAS. The network consists of 10 observation nodes. The longitude, latitude and elevation of the nodes are given as follows.  01: 125.621667°E, 44.791753°N; 187 m  02: 125.621878°E, 44.791487°N; 186 m  03: 125.622080°E, 44.791242°N; 186 m  04: 125.622249°E, 44.791028°N; 186 m  05: 125.622407°E, 44.790820°N; 187 m  06: 125.622000°E, 44.791875°N; 188 m  07: 125.622206°E, 44.791603°N; 187 m  08: 125.622389°E, 44.791350°N; 187 m  09: 125.622564°E, 44.791128°N; 187 m  10: 125.622736°E, 44.790917°N; 187 m | The study area is in a mid-temperate continental monsoon climate, with an annual average temperature of 4.4°C, and an annual average precipitation of 520 mm. The area is in the core corn belt of the Jilin Province. Only one crop is planted every year, and the crop growth period is from May to October. The crop types in 2020 and 2021 are corn.  The soil type where the soil sensor is installed is silty loam, with 10.27% clay, 53.18% silt, and 36.55% sand. The contents of soil organic matter (SOM) and soil salinity is 2.66% and 0.034%, respectively. |
| GuYuan  （GY） | GuYuan network is in the area north of Guyuan County, Zhangjiakou city, Hebei Province, which is 12 km far from Guyuan County. The network is at the China Agricultural University Guyuan experimental station. The network consists of 10 observation nodes. The longitude, latitude and elevation of the nodes are given as follows.  01: 115.680614°E, 41.763509°N; 1386 m  02: 115.681017°E, 41.763448°N; 1386 m  03: 115.681394°E, 41.763378°N; 1386 m  04: 115.681325°E, 41.763076°N; 1386 m  05: 115.681237°E, 41.762806°N; 1386 m  06: 115.680913°E, 41.762857°N; 1386 m  07: 115.680553°E, 41.762901°N; 1386 m  08: 115.680734°E, 41.763017°N; 1386 m  09: 115.680962°E, 41.763137°N; 1386 m  10: 115.680571°E, 41.763203°N; 1386 m | The study area is in the central region of the Bashang Plateau, which is a typical grassland area with a semiarid continental monsoon climate. Annual precipitation is between 350-450 mm, with an average temperature of approximately 1°C. Located in the northern Hebei Province, this area is part of the ecotone of agriculture and animal husbandry. The growing season is from May to October. The crop from June 2019 to August 2021 is Leymus chinensis.  The soil in which the sensors are installed is chestnut soil with high potassium content, medium nitrogen content and low phosphorus content. The soil type where the soil sensor is installed is silty loam, with 7.20% clay, 37.13% silt, and 55.67% sand. The content of SOM and soil salinity were 2.36% and 0.2789%, respectively. |
| HeFei  (HF) | HeFei network is in the suburb of the Shushan District, Hefei City, Anhui Province. The network is in the experimental area of the Hefei Institutes of Physical Science, CAS. The network consists of 10 observation nodes. The longitude, latitude and elevation of the nodes are given as follows.  01: 117.169620°E, 31.903757°N; 26 m  02: 117.169769°E, 31.903918°N; 26 m  03: 117.169786°E, 31.903749°N; 26 m  04: 117.169930°E, 31.903913°N; 26 m  05: 117.169931°E, 31.903758°N; 26 m  06: 117.169656°E, 31.903428°N; 26 m  07: 117.169834°E, 31.903293°N; 26 m  08: 117.169817°E, 31.903423°N; 26 m  09: 117.169948°E, 31.903285°N; 26 m  10: 117.169942°E, 31.903423°N; 26 m | The study area is in a subtropical humid monsoon climate, with an annual average temperature of 15.7°C, and an annual average precipitation of 1000 mm. The experimental area is in the suburb of Hefei city. The vegetation types in April 2019 are grasses and sparse, low plants. The soil type where the soil sensor is installed is yellow‒brown earth, with 11.22% clay, 81.28% silt, and 7.50% sand. The content of SOM and soil salinity were 1.89% and 0.047%, respectively. |
| HaiBei  (HB) | HaiBei network is in Fengxiakou village, Menyuan Hui Autonomous County, Tibetan Autonomous Prefecture of Haibei, Qinghai Province. The network is in the experimental area of the Qinghai Haibei remote sensing test station, CAS. The network consists of two sample regions, each of which contains five observation nodes. The longitude, latitude, and altitude of each node are given as follows.  HaiBei1：  01: 101.313030°E, 37.610811°N; 3198 m  02: 101.313268°E, 37.610823°N; 3198 m  03: 101.313157°E, 37.610741°N; 3198 m  04: 101.313062°E, 37.610656°N; 3198 m  05: 101.313275°E, 37.610665°N; 3198 m  HaiBei2：  06: 101.312261°E, 37.611732°N; 3201 m  07: 101.312193°E, 37.611630°N; 3201 m  08: 101.312128°E, 37.611546°N; 3201 m  09: 101.312052°E, 37.611450°N; 3201 m  10: 101.311979°E, 37.611353°N; 3201 m | The study area is in a plateau continental monsoon climate, with an annual average temperature of -1.7°C and an annual average precipitation of 580 mm. The entire experimental area was covered with herbage, and the dominant plant populations are Kobresia humilis, Stipa aliena, Elymus nutans, and Gentiana farreri. The vegetation types in 2019 and 2021 are the vegetation described above. The soil type where the soil sensor is installed is silty loam, with 7.29% clay, 82.83% silt, and 9.88% sand. The content of SOM and soil salinity were 17.46% and 0.014%, respectively. |
| JiangShanJiao  (JSJ) | JiangShanJiao network is in Jiangshanjiao Experimental Forest Farm, Jingpo Town, Ning'an City, Mudanjiang City, Heilongjiang Province. The network is in the experimental area of the Heilongjiang Forest Industry Group. The network consists of 10 observation nodes. The longitude, latitude and elevation of the nodes are given as follows.  01: 128.952030°E, 43.855463°N; 421 m  02: 128.952123°E, 43.855404°N; 422 m  03: 128.952224°E, 43.855342°N; 423 m  04: 128.952316°E, 43.855288°N; 423 m  05: 128.952400°E, 43.855232°N; 424 m  06: 128.952177°E, 43.855559°N; 422 m  07: 128.952274°E, 43.855495°N; 422 m  08: 128.952362°E, 43.855435°N; 426 m  09: 128.952448°E, 43.855377°N; 424 m  10: 128.952531°E, 43.855243°N; 425 m | The study area is in a mid-temperate continental monsoon climate, with an annual average temperature of 4.5°C and an annual average precipitation of 550 mm. The experimental area is covered in grassland. The soil type where the soil sensor is installed is clay soil. The contents of SOM and soil salinity were 10.28% and 0.008%, respectively. |
| XiTianShan  (XTS) | XiTianShan network is in the "Science and Technology Demonstration Base and industry-University-Research Collaborative Innovation Base of Yili Normal University-Yili Forestry Research Institute" in Qapqal Xibe Autonomous County, Yili Kazak Autonomous Prefecture, Xinjiang. The network consists of 10 observation nodes. The longitude, latitude and elevation of the nodes are given as follows.  01: 81.172565°E, 43.744352°N; 735 m  02: 81.172557°E, 43.744132°N; 735 m  03: 81.172552°E, 43.743942°N; 735 m  04:81.172551°E, 43.743757°N; 735 m  05:81.172541°E, 43.743570°N; 735 m  06:81.172536°E, 43.743383°N; 735 m  07:81.172531°E, 43.743201°N; 735 m  08:81.172526°E, 43.743038°N; 735 m  09:81.172521°E, 43.742860°N; 735 m  10:81.172518°E, 43.742681°N; 735 m | The study area has a temperate and arid climate in the north continental temperate zone, with an annual average temperature of 7.9°C and an annual average precipitation of 222 mm.  The crop in the experimental area is apple, which was planted in 2015.  The soil type where the soil sensor is installed is silty loam, with 6.69% clay, 58.85% silt, and 34.46% sand. The contents of SOM and soil salinity were 2.35% and 0.108%, respectively. |
| MinQin  (MQ) | MinQin network is in the Minqin Integrated Desert Control Experiment station, Xuebai Town, Minqin County, Wuwei City, Gansu Province. The network is in the experimental area of the Gansu Minqin National station for Desert Steppe Ecosystem Studies, Gansu Desert Control Research Institute. The network consists of 10 observation nodes. The longitude, latitude and elevation of the nodes are given as follows.  01: 102.917692°E, 38.629643°N; 1375 m  02: 102.917884°E, 38.629393°N; 1375 m  03: 102.918092°E, 38.629149°N; 1375 m  04: 102.917985°E, 38.629801°N; 1375 m  05: 102.918207°E, 38.629532°N; 1375 m  06: 102.918403°E, 38.629301°N; 1375 m  07: 102.918277°E, 38.629765°N; 1375 m  08: 102.918302°E, 38.629966°N; 1375 m  09: 102.918538°E, 38.629723°N; 1375 m  10: 102.918711°E, 38.629474°N; 1375 m | The study area is in a temperate continental desert climate, with an annual average temperature of 7.6°C and an annual average precipitation of 113.8 mm. The trial was conducted in the desert area located at the fringe of the Minqin oasis. The vegetation is dominated by sparse natural *Nitraria tangutorum Bor.* communities. The growing season is from April to October.  The soil type where the soil sensor is installed is sand and loamy sand, with 1.34% clay, 10.72% silt, and 87.94% sand. The contents of SOM and soil salinity were 0.18% and 0.049%, respectively. |
| HuLunBeiEr  (HLBE) | HuLunBeiEr network is in the Xieertala, Hailar District, Hulunber City, Inner Mongolia Autonomous Region. The network is in the experimental area of the National Field Scientific Observation and Research station of Hulunber Grassland Ecosystem in Inner Mongolia. The network consists of 10 observation nodes. The longitude, latitude and elevation of the nodes are given as follows.  01: 119.988784°E, 49.332319°N; 635 m  02: 119.989215°E, 49.332378°N; 635 m  03: 119.989606°E, 49.332428°N; 634 m  04: 119.988905°E, 49.332014°N; 635 m  05: 119.989724°E, 49.332125°N; 635 m  06: 119.989576°E, 49.331946°N; 635 m  07: 119.989384°E, 49.331791°N; 635 m  08: 119.989785°E, 49.331845°N; 635 m  09: 119.989313°E, 49.332077°N; 635 m  10: 119.989028°E, 49.331747°N; 635 m | The precipitation and temperature in the study area are moderate, and the climate is temperate continental. The annual precipitation generally averages 300 mm to 400 mm and is mainly concentrated in June to September. The frost-free period is almost 110 days, and the mean annual air temperature is −4°C to 1°C. The soil type where the soil sensor is installed is chernozem soil with an intermediate fertility level. The type of land cover in the study area is grassland. The soil type where the soil sensor is installed is sand and loamy sand, with 11.32% clay, 57.06% silt, and 31.62% sand. The contents of SOM and soil salinity were 8.03% and 0.032%, respectively. |
| XiLinHaoTe  (XLHT) | XiLinHaoTe network is in Mauden ranch, Xilinhot, Xilingol League, Inner Mongolia, China. The network is in the experimental area of the Xilinhot National Climate Observatory test station, China Meteorological Administration. The network consists of 10 observation nodes. The longitude, latitude and elevation of the nodes are given as follows.  01: 116.329881°E, 44.136899°N; 1100 m  02: 116.330233°E, 44.136924°N; 1100 m  03: 116.330591°E, 44.136932°N; 1100 m  04: 116.329919°E, 44.136643°N; 1100 m  05: 116.330262°E, 44.136660°N; 1100 m  06: 116.330581°E, 44.136674°N; 1100 m  07: 116.330104°E, 44.136525°N; 1100 m  08: 116.329954°E, 44.136405°N; 1100 m  09: 116.330290°E, 44.136413°N; 1100 m  10: 116.330587°E, 44.136425°N; 1100 m | The study area is in a mid-temperate continental monsoon climate, with an annual average temperature of 3.1°C, and an annual average precipitation of 263.5 mm. The experimental area is in the core belt of Inner Mongolia's Xilingol League. The underlying surface is natural grassland, and the growing period of natural herbage is from April to October. The soil type where the soil sensor is installed is loam, with 8.35% clay, 37.15% silt, and 54.45% sand. The contents of SOM and soil salinity were 3.32% and 0.06%, respectively. |
| QiYang  (QY) | QiYang network is in Guanshanping village, Wenfushi Township, Qiyang County, Hunan Province. The network is in the experimental area of Hengyang Red Soil Experimental station, Chinese Academy of Agricultural Sciences. The network consists of 10 observation nodes. The longitude, latitude and elevation of the nodes are given as follows.  01: 111.871059°E, 26.760063°N; 150 m  02: 111.871058°E, 26.759924°N; 151 m  03: 111.871048°E, 26.759790°N; 151 m  04: 111.870934°E, 26.759923°N; 151 m  05: 111.871046°E, 26.759676°N; 150 m  06: 111.871180°E, 26.760193°N; 148 m  07: 111.871170°E, 26.760066°N; 149 m  08: 111.871164°E, 26.759924°N; 150 m  09: 111.871158°E, 26.759791°N; 149 m  10: 111.871153°E, 26.759671°N; 149 m | The climate of the study area is classified as subtropical humid monsoon with average annual temperature of 18°C, annual precipitation of 1310 mm, sunshine duration is 1613 hours and solar radiation is 4370 MJ/m^2^. The frost-free period is approximately 300 days. The soil type where the soil sensor is installed is red earth developed from Quaternary red clay, with 16.79% clay, 74.24% silt, and 8.9% sand. The content of SOM is 1.66%. The soil pH is 4.2. The crop at the observation plot is perennial evergreen tea trees planted 40 years ago. Tea tree is one of main cash crops in the hilly red soil region of Southern China. |
| DongTingHu  (DTH) | DongTingHu network is in Kangwang village, Nong'an County, Yueyang City, Hunan Province. The network is in the Yueyang Academy forest. The network consists of 10 observation nodes. The longitude, latitude and elevation of the nodes are given as follows.  01: 113.168016°E, 29.315266°N; 53 m  02: 113.168121°E, 29.315338°N; 53 m  03: 113.168105°E, 29.315232°N; 53 m  04: 113.168081°E, 29.315161°N; 53 m  05: 113.168188°E, 29.315214°N; 53 m  06: 113.168425°E, 29.315000°N; 57 m  07: 113.168648°E, 29.315061°N; 57 m  08: 113.168842°E, 29.315133°N; 57 m  09: 113.169053°E, 29.315196°N; 57 m  10: 113.169272°E, 29.315275°N; 57 m | The study area is in a subtropical monsoon climate, with an annual average temperature of 4.4°C and an annual average precipitation of 1350 mm. The experimental area is in the Camellia oleifera and paddy rice belt of the Hunan Province. Double cropped rice can be planted every year, and the crop growth period is from May to October. The crop types in 2020 and 2021 are Camellia oleifera. The soil type where the soil sensor is installed is silty loam, with 13.02% clay, 81.78% silt, and 5.20% sand. The content of SOM and soil salinity were 2.67% and 0.12%, respectively. |
| GuangZhou  (GZ) | GuangZhou network is in Fengcun village, Xintang Town, Zengcheng County, Guangzhou City, Guangdong Province. The network is in the experimental area of South China Agricultural University. The network consists of 10 observation nodes. The longitude, latitude and elevation of the nodes are given as follows.  01: 113.633850°E, 23.244480°N; 21 m  02: 113.634052°E, 23.244852°N; 22 m  03: 113.634172°E, 23.245119°N; 23 m  04: 113.634277°E, 23.245308°N; 24 m  05: 113.634294°E, 23.244958°N; 23 m  06: 113.634365°E, 23.245105°N; 23 m  07: 113.634205°E, 23.244328°N; 21 m  08: 113.634386°E, 23.244678°N; 22 m  09: 113.634530°E, 23.244981°N; 23 m  10: 113.634627°E, 23.245161°N; 24 m | The study area belongs to the subtropical marine monsoon climate, with an annual average temperature of 21.6°C, and an annual average precipitation of 1820 mm. The experimental area is located at the experiment station and agriculture training centre of South China Agricultural University, and the area is planted with lawn grass in 2020 and 2021. The soil type where the soil sensor is installed is clay loam, and the surface is covered with sand to facilitate the planting of lawn grass. The soil type where the soil sensor is installed is sand and loamy sand, with 5.36% clay, 55.58% silt, and 39.05% sand. The contents of SOM and soil salinity were 1.42% and 0.041%, respectively. |
| YuCheng  (YC) | YuCheng network is in Yuju Road, Yucheng City, Dezhou City, Shandong Province. The network is in the experimental area of Shandong Yucheng remote sensing test station, CAS. The network consists of 10 observation nodes. The longitude, latitude and elevation of the nodes are given as follows.  01: 116.570341°E, 36.828925°N; 23 m  02: 116.570262°E, 36.828727°N; 23 m  03: 116.570200°E, 36.828533°N; 23 m  04: 116.570121°E, 36.828343°N; 23 m  05: 116.570069°E, 36.828173°N; 23 m  06: 116.569745°E, 36.828229°N; 23 m  07: 116.569802°E, 36.828403°N; 23 m  08: 116.569867°E, 36.828593°N; 23 m  09: 116.569925°E, 36.828770°N; 23 m  10: 116.570001°E, 36.828965°N; 23 m | The study area is in a semihumid monsoon climate zone in the warm temperate zone, with an average annual temperature of 4.2°C and an average annual rainfall of 460 mm. The experimental area is in the corn core belt of the Shandong Province. Two crops are planted every year. The crop types in 2020 and 2021 are corn and wheat. The type of soil where the soil sensor is installed is silty loam, in which clay content is 7.07%, silt content is 79.12%, and sand content is 13.81%. The SOM and salinity content are 1.75% and 0.011%, respectively. |
| NanJing  (NJ) | NanJing network is in Yuxiang village, Shangxing Town, Liyang City, Jiangsu Province. The network is in the test area of Nanjing station, AIR, CAS. The network consists of 10 observation nodes. The longitude, latitude and elevation of the nodes are given as follows.  01: 119.212802°E, 31.502421°N; 18 m  02: 119.213283°E, 31.502218°N; 18 m  03: 119.212703°E, 31.502261°N; 18 m  04: 119.212946°E, 31.502144°N; 18 m  05: 119.213186°E, 31.502047°N; 17 m  06: 119.212606°E, 31.502086°N; 18 m  07: 119.212844°E, 31.501986°N; 18 m  08: 119.213086°E, 31.501884°N; 17 m  09: 119.212508°E, 31.501896°N; 19 m  10: 119.212981°E, 31.501706°N; 18 m | The study area is in a north subtropical monsoon climate, with an annual average temperature of 15.5°C and an annual average precipitation of 1152.1 mm. The experimental area is in the core of the tea gardens in the Jiangsu Province. Only one crop is planted every year, and the growth period is from April to October. The crop type in 2020 and 2021 is tea. The soil type where the soil sensor is installed is sand and loamy sand, with 6.61% clay, 61.43% silt, and 31.96% sand. The content of SOM and soil salinity were 1.35% and 0.087%, respectively. |
| QingDao  (QD) | QingDao network is in the Huangdao District, Qingdao city, Shandong Province. The network is at the China University of Petroleum (East China) campus. The network consists of two sample regions, each of which contains five observation nodes. The longitude, latitude, and altitude of each node are given as follows.  QingDao1：  01: 120.176894°E, 35.946467°N; 22 m  02: 120.177224°E, 35.946487°N; 16 m  03: 120.177065°E, 35.946336°N; 19 m  04: 120.176915°E, 35.946212°N; 16 m  05: 120.177233°E, 35.946235°N; 16 m  QingDao2：  06: 120.172490°E, 35.944666°N; 3 m  07: 120.172700°E, 35.944791°N; 3 m  08: 120.172674°E, 35.944638°N; 3 m  09: 120.172648°E, 35.944496°N; 3 m  10: 120.172832°E, 35.944630°N; 3 m | The study area is in a monsoon climate of medium latitude, with an annual average temperature of 12.7°C, and an annual average precipitation of 662.1 mm. This experimental area is in the coastal area of the Shandong Province and has a remarkable maritime climate. The crops in experimental area 1 are pine and fruit trees. The crops in experimental area 2 are grassland. The soil type where the soil sensor is installed is sand and loamy sand, with 7.08% clay, 54.87% silt, and 38.05% sand. The content of SOM and soil salinity were 1.32% and 0.145%, respectively. |
| QianYanZhou  (QYZ) | QianYanZhou network is in Guanxi Town, Taihe County, Ji’an City, Jiangxi Province. The network is in the experimental area of the Qiangyanzhou Ecological station, CAS. The network consists of 10 observation nodes. The longitude, latitude and elevation of the nodes are given as follows.  01: 115.071520°E, 26.745112°N; 73 m  02: 115.071803°E, 26.745190°N; 72 m  03: 115.071735°E, 26.745031°N; 73 m  04: 115.071665°E, 26.744896°N; 72 m  05: 115.071897°E, 26.744960°N; 72 m  06: 115.072050°E, 26.745256°N; 72 m  07: 115.072339°E, 26.745356°N; 72 m  08: 115.072253°E, 26.745178°N; 72 m  09: 115.072146°E, 26.745019°N; 72 m  10: 115.072447°E, 26.745118°N; 72 m | This area is in a subtropical continental monsoon climate, with an annual average temperature of 17.9°C, and an annual average precipitation of 1505 mm. This experimental area is in the hilly area in the Ji-Tai basin of the Jiangxi Province. Only one shrub type (*Broussonetia papyrifera* hybrid cultivar) is planted, and it is harvested five times during growth period every year (May to October). The soil type where the soil sensor is installed is typical red soil, and it is classified as Ultisols. The SOM is 8.8 g·kg^–1^, with bulk density (0–20 cm) of 1.50 g·cm^-3^, pH of 4.90, and total N content of 0.7 g·kg^–1^. |
| NaQu  (NQ) | NaQu alpine grassland ecosystem observation station is in Quguorenmao village, Naqu Town, Seni County, Naqu City, Xizang Province. The network is at the Institute of Geographic Sciences and Natural Resources Research, CAS. The network consists of 10 observation nodes. The longitude, latitude and elevation of the nodes are given as follows.  01: 92.011623°E, 31.643536°N; 4598 m  02: 92.011906°E, 31.643489°N; 4598 m  03: 92.012156°E, 31.643452°N; 4598 m  04: 92.011587°E, 31.643214°N; 4598 m  05: 92.011857°E, 31.643176°N; 4598 m  06: 92.012100°E, 31.643144°N; 4598 m  07: 92.011954°E, 31.643028°N; 4598 m  08: 92.011559°E, 31.642935°N; 4598 m  09: 92.011809°E, 31.642915°N; 4598 m  10: 92.012046°E, 31.642892°N; 4598 m | The study area is in an alpine subfrigid zone with monsoon subhumid climate, with an annual average temperature of -2.8~-1.6°C, and an annual average precipitation of 247.3~513.6 mm. The experimental area is covered by typical alpine grassland, and the dominant species is *Kobresia myosuroides*. The soil type where the soil sensor is installed is alpine meadow soil. In addition, the soil type is sand and loamy sand, with 10.50% clay, 84.20% silt, and 5.29% sand. The content of SOM and soil salinity were 1.37% and 0.076%, respectively. |
